# The impact of social variables in preclinical models of cocaine abuse

**DOI:** 10.12703/r/10-76

**Published:** 2021-10-18

**Authors:** Michael A Nader

**Affiliations:** 1 Department of Physiology and Pharmacology, Wake Forest School of Medicine, 546 NRC, Medical Center Boulevard, Winston-Salem, NC 27157-1083, USA

**Keywords:** Animal Models, Social Rank, Social Reward, Social Modelling, Cocaine, Self-Administration

## Abstract

At present, there are no US Food and Drug Administration–approved treatments for cocaine use disorders. One consideration for this lack of treatment efficacy stems from the appropriate use of animal models. The premise of this commentary is that social behavior needs to be incorporated in animal models of cocaine use disorder. The goal of this commentary is to describe some of the strengths and limitations of recent preclinical animal models of cocaine abuse which have incorporated social behavior. There are many ways to include social variables into preclinical research, and the study design will depend on the questions asked. Four general types of studies incorporating social factors are described: those involving aggression (that is, maternal neglect and social defeat), modeling, social reward, and social housing, including social isolation. The inclusion of social variables into preclinical research will help identify biobehavioral markers that may lead to an individualized treatment approach that more effectively decreases cocaine use. These studies will aid in the development of novel pharmacotherapies as well as non-pharmacological interventions (for example, punishment, alternative reinforcers, and environmental enrichment) that would be critical for informing policy decisions.

## Introduction

The focus of this commentary will be on the use of preclinical animal models of cocaine abuse, and the emphasis will be on social behavior. Translational research, as described by the National Institutes of Health (NIH), is a two-way street. Basic scientists provide clinicians with new tools to treat disease, and clinical researchers make novel observations (about the nature and progression of disease) that can lead to innovative basic science investigations. For animal researchers to truly provide impactful information to clinicians, social behavior needs to be a component of the model. In a theoretical paper, Garner^1^ stated: “we need to do a better job of producing animal results that translate to human outcomes. The real challenge, however, is changing the culture of biomedical research so that even small simple changes are adopted. The potential rewards are worth the effort. At the end of the day, the failure of animal results to translate is arguably the greatest laboratory animal welfare issue of our day and a source of many societal ills” (p. 439). The same has been made for the neuroscience of addiction^2–4^. Heilig *et al.*^3^ noted that incorporating social factors into animal models of addiction would be important for discovering new treatments. Because there have been many excellent reviews on this topic^5–8^, the focus of this commentary will be on experimental design considerations involving socially housed animals. Two further noteworthy features of the studies will be described: they will involve only models of drug self-administration, and the review will be limited to studies involving cocaine. Drug self-administration procedures require the animal to make responses that will result in the delivery of the drug. An important independent variable in drug self-administration studies is the schedule of reinforcement—or the parameters (for example, number of responses and passage of specific periods of time) that determine when a response results in the drug delivery (termed the contingency). For the studies described in this commentary, cocaine was always delivered intravenously. The primary dependent variables in self-administration studies are typically the number of drug injections, response rates as a function of drug dose, total drug intake, and “other” behaviors (food-maintained responding, social interactions, and so on).

There are many considerations when studying the influence of social variables on the reinforcing effects of cocaine. For example, what’s the comparator group, individually housed animals? Is the hypothesis that social housing is an environmental enricher, similar to studies in which food is the alternative to cocaine? Is social status an important consideration, and is the study designed such that social rank is an independent or a dependent variable? Will the impact of social conditions be different depending on the stage of the addiction cycle (initiation, maintenance, abstinence, or relapse)? When a researcher decides to incorporate social variables into the experimental design, there are multiple ways these studies could be conducted. For this commentary, four general types of studies related to the effects of social variables on cocaine self-administration will be briefly described:

1. Aggression involving maternal neglect and social defeat
2. Modeling
3. Social reward
4. Social housing.

There is no single way to incorporate social variables into a preclinical study, and how social factors are incorporated will depend on the questions being asked by the investigator. Is the social context an antecedent condition, part of the current environmental context, or a maintaining event? For example, most intravenous drug self-administration studies require individually housed animals, at least during the period of self-administration. In one type of study design, these animals could be “challenged” with a social confrontation to examine how defeat or aggression or both impact drug use. Other studies have put animals in the context of another animal, their peer, to examine how the behavior of the other animal impacts cocaine self-administration or make access to another animal a consequence of not choosing to self-administer cocaine. Finally, the animals could be socially housed, and the social status of the animal serves as an independent variable that may influence drug self-administration or treatment outcomes or both. While these are all examples of incorporating social variables into cocaine self-administration studies, each design addresses a different question and therefore will lead to different conclusions.

These are important considerations for preclinical researchers. As stated by Bardo *et al.*^5^, “the vast majority of preclinical studies in this area have examined either individual differences or social influences as separate independent predictors of sensitivity to abused drugs. Although this approach has yielded predictive relations and key neural mechanisms, it does not capture the multifaceted nature of drug abuse vulnerability in humans. A major challenge will be to identify what combination of individual differences and different social contexts influences the neurobehavioral pharmacology of abused drugs. Moreover, the interactive effects of individual and social-based differences remain an understudied area” (p. 281).

## Social variables involving aggression

### Maternal aggression and adolescent maltreatment

There are several considerations involving social aggression, including when the aggressive encounters occur (that is, early in life or during adulthood). For example, as with humans, infant maltreatment, characterized by aggression of the mother toward her infant^9^, occurs spontaneously in nonhuman primates and with similar prevalence rates as in humans^10^. Thus, in this model, the early history of abuse can be studied for their long-term consequences on hormones^11^, brain function^12,13^, behavior^14^, and drug self-administration^15,16^. In two recent cocaine self-administration studies involving adolescent female and male rhesus monkeys, half of whom were maltreated as infants, the effects on acquisition and maintenance of cocaine self-administration were examined^15,16^. In the first study^15^, previously cocaine-naïve rhesus monkeys self-administered 0.03 mg/kg per injection cocaine under a fixed-ratio (FR) 20 schedule of reinforcement. This contingency requires 20 responses on a lever (the operant) to deliver cocaine intravenously. Acquisition was defined as five consecutive sessions in which monkeys earned 19 or 20 injections per session. Maltreated monkeys acquired faster than control monkeys, suggesting that early life stress enhanced the sensitivity to cocaine as a reinforcer. In the follow-up study from this group, the ability to show escalation of cocaine self-administration, a phenomenon in which the number of cocaine injections increases with continued exposure and which has not previously been demonstrated in nonhuman primates, was examined by increasing the session length from 1 to 4 hours. The hypothesis was that the early life stress may increase the likelihood that these monkeys would show escalation of cocaine self-administration. No escalation was noted in the maltreated or control monkeys^16^. However, it remains to be determined whether medication or behavioral treatment efficacy and brain changes during cocaine abstinence are influenced by this early life experience. Several rodent models also explore this early life stress, primarily from the perspective of social isolation, and those will be described in the last section. These models provide critical information related to the impact of early life stress on subsequent behavior, including maladaptive behavior. More research needs to focus on this population, including the incorporation of prenatal drug exposure on subsequent behavior (including social behavior).

### Social defeat/intruder paradigms

Another form of social influence that is proximate to the cocaine self-administration session involves the study of social defeat using a resident–intruder paradigm^17,18^. In this paradigm, primarily studied in male rodents (for female rats, see 19), an animal (the intruder) is placed into the home cage of another, aggressive animal (the resident). Not surprisingly, acute social stress from being the intruder resulted in increased acquisition of cocaine self-administration and increased sensitivity to the reinforcing effects of cocaine^20^. Rather than review the literature using this paradigm, I want to convey the message that this experimental manipulation (making an animal an intruder) does not necessarily produce the same outcomes in all subjects. Rather than consider the outcomes as equivocal, we should study these individual differences. For example, Wood *et al.*^21^ exposed male Long–Evans rats to five consecutive 30-minute intruder conditions. The primary behavioral dependent variable was average defeat latencies— or the time to exhibit a supine submissive posture. They identified two phenotypes—rats exhibited either a “passive coping strategy”, in which there was a short latency to assume a defeat posture, or an “active coping strategy” characterized by longer latencies to submit. Of significance was that the coping strategy was associated with distinct gene expression changes: passive rats showed increases in proinflammatory processes, whereas rats in the active coping group showed gene expression changes associated with suppression of inflammatory processes. The investigators suggest that this may be one mechanism to account for individual differences in stress-related pathologies.

A recent study in monkeys extended these findings of individual differences to include the influence of social status on behavioral and neuronal consequences to being an intruder^22^. In that study, male monkeys lived in social groups of four per pen and a linear social hierarchy was established (#1 dominant to #4 subordinate). These monkeys were placed in operant chambers and self-administered intravenous cocaine daily under a concurrent choice schedule of reinforcement in which the alternative was a banana-flavored food pellet. To examine the effects of social stress, using the resident–intruder paradigm, either the #1- or #4-ranked monkey was removed from their social group and placed in one quadrant (the lower left quadrant) of another social group of four male monkeys; the intruder was physically protected from direct contact with the other four monkeys, who were on top and around him. Two separate experiments were conducted with the intruder: (1) the effects of acute social stress were examined on cocaine vs. food choice and (2) brain glucose utilization with [^18^F]fluorodeoxyglucose and positron emission tomography (PET) imaging. As it relates to cocaine self-administration, being an intruder increased sensitivity to the reinforcing effects of cocaine in subordinate monkeys, as hypothesized (that is, the effects of stress should shift the cocaine dose-response curve to the left). Surprisingly, that same manipulation decreased cocaine self-administration in dominant monkeys (that is, shifted the cocaine dose-response curve to the right)—a pattern that resembles the effects of environmental enrichment on cocaine self-administration^23^. In terms of potential mechanisms, brain glucose metabolism in their home cage and following being the intruder was different for dominant and subordinate monkeys. Consistent with the different coping strategies described by Wood *et al.*^21^, how the monkeys responded to being the intruder also showed rank-related differences: dominant monkeys showed significantly higher levels of aggression compared with subordinate monkeys, even though they were both intruders in another social group. Aggression can function as a reinforcer in both rats^24,25^ and mice^26,27^. One hypothesis is that the stress-induced aggression was actually enriching to dominant monkeys and that may account for the rightward shift in the cocaine dose-response curve. Irrespective of the behavioral or neuronal mechanisms, the phenotypic differences in response to apparently similar environmental events support a personalized treatment approach to cocaine use disorders.

## Modeling

As described by Strickland and Smith^28^, models of social learning propose that attitudes and behaviors held by a group are transmitted to its individual members and thereby influence behavior. In a pioneering study, Smith^29^ examined three groups of pair-housed rats in which intravenous cocaine self-administration was evaluated in custom-built, operant conditioning chambers. These operant chambers allowed two rats to have visual proximity to each other. The relevant groups were the following: (1) both rats had access to cocaine and, as a result, each rat could see the other one self-administering cocaine; (2) one rat had access to cocaine while the other rat had a lever in the chamber but did not have access to cocaine; and (3) an individually housed rat with access to cocaine (that is, the standard laboratory condition). Smith reported that self-administration was highest among rats whose social partner was also self-administering cocaine. Just as importantly, the lowest rates of self-administration occurred in rats in which they had a social partner that did not self-administer cocaine.

Strickland and Smith^28^ discuss several possible explanations for the high rates of self-administration in the rat that had a social partner who was also self-administering cocaine. These possibilities include social reinforcement, social facilitation, peers as discriminative stimuli, and peers as conditioned stimuli. The most important point about these potential mechanisms is that treatment strategies that do not incorporate factors related to peer influence will most likely be unsuccessful. Furthermore, it may be the case that incorporating the peer into the treatment evaluation will enhance outcome. For example, what if drug access for the peer changed from cocaine to a non-drug reinforcer, like food pellets, or that animal had a choice and cocaine self-administration decreased? That may decrease subsequent cocaine self-administration in the peer but probably not eliminate it. An important observation from these studies is that the group in which the peer did not have access to cocaine still self-administered cocaine, just at lower rates compared to rats in which both pairs had access to cocaine. From a harm reduction standpoint, that is a good outcome, but perhaps the addition of a behavioral (for example, exercise) or a pharmacological agent would further decrease cocaine use. It is also an empirical question that changing conditions for the peer will change self-administration for the cage mate. Such studies are certainly worth conducting.

## Social reward

The field of behavioral pharmacology has a long history of studying cocaine self-administration in the context of alternative non-drug reinforcers, primarily food reinforcers (for example, 30,31). One of the first examples of social reward in the context of cocaine reward involved models of maternal behavior using conditioned place preference (CPP) procedures in which female rats had to choose between their dams and cocaine (for example, 32). CPP incorporates conditioned stimuli, based on Pavlovian principles, in which one side of a chamber is associated with one stimulus (for example, the dam’s pups) and the other side is associated with a non-contingent injection of cocaine (see 33 for advantages and disadvantages of CPP). More recently, Shaham *et al.* used operant procedures, concurrent schedules, to study choice between access to an intravenous injection of drug or access to a social partner^34^. These investigators recently published detailed methods^35^, so a full description of the procedure will not be repeated here. Instead, this section will focus on additional questions that can be addressed under these drug vs. social access contingencies.

In the original study^34^, rats were initially pair-housed; one rat eventually served as the self-administration rat and the other one served as the social partner. At the start of the experiment, rats were trained to respond for access to a social partner. The session started with illumination of a house light, which served as a discriminative stimulus. Responding was maintained under an FR 1 schedule of reinforcement, with a 60-second limited hold (LH) (a response had to be made within 60 seconds of house-light illumination) with completion of that response resulting in an extinguishing of the house light, retraction of the lever, presentation of a 20-second tone (this would serve as a conditioned stimulus, CS+, by being paired with the social partner) followed by the opening of the guillotine-style sliding door. The resident rat was permitted to interact with their social partner for 60 seconds until the guillotine door closed. After six sessions of social-access training, rats were implanted with an indwelling intravenous catheter and trained under a similar procedure (FR 1, LH 60 seconds) for intravenous injections of drug (either methamphetamine or heroin in the initial study). So that “voluntary abstinence” (see 36) could be studied, rats were given a choice between social access and intravenous drug administration. Under these conditions, rats chose social access almost exclusively, hence the descriptor “voluntary abstinence”. Importantly, social reward behavior responded in an orderly fashion to increases in response requirement (for example, progressive-ratio responding requires an increase in the number of responses to gain access to a social partner), delays, and punishment^8,34^.

Although these are exciting studies and a very promising animal model of human drug-taking, there are a few observations to consider. First, as described by the investigators, “we manually replaced both rats in their appropriate chambers” at the end of a social access trial (34, p. 8). By definition, a free operant refers to automated conditions that “free” the investigator from being involved during experimental sessions. As designed, this procedure is not a free operant. As a follow-up, these investigators established an automated system for studying social behavior, although direct physical contact was limited by a screen^37^. One possibility to consider for future studies is to use conditioned stimuli to maintain a larger number of responses. A related point is that these studies involve multiple discrete-trial choice procedures. It would be interesting to extend these studies to a mutually exclusive choice with only one trial—they choose either drug or access to a social partner. This would now certainly become a free operant, and the use of conditioned stimuli could result in more behavior than would be observed under standard discrete-trial choice procedures. Another consideration for the model, taken from food-drug choice procedures, involves the question of how to alter the magnitude of the alternative, non-drug (that is, social) reinforcer. Would this involve manipulating the number of rats involved in the social contact or the duration of access to a social partner? Would these two manipulations produce similar results? What about social status? Do the rats in the self-administration condition have to be familiar with the rats used as social rewards? If not, is that tapping into another type of social condition? What about sex differences? These are exciting questions for future research.

## Social housing

In this section, I briefly describe the two extremes of “social housing”: isolation and group housing. Social isolation may be considered a model of stress that could translate to human conditions involving social exclusion generated by racism and discrimination^4^. In a recent review of rodent models^38^, much of the ongoing research uses adolescent social isolation and the effects on adult behavior and neurobiology. Noschang *et al.*^38^ noted only five studies involving adolescent social isolation and subsequent adult cocaine self-administration. In all cases, the consequences were higher rates of cocaine self-administration in the isolation group compared with group-housed rodents also tested in adulthood (see Table 1 in 38). Of the five studies described, three of them used progressive-ratio schedules of reinforcement^39–41^; following a cocaine injection, the response requirement increased to receive the next injection. Progressive-ratio schedules of reinforcement provide a better measure of reinforcing strength than simple schedules of reinforcement (for example, FR 1). Of course, the issue for social isolation studies is the comparator group, which is typically group-housed subjects. As a result, it is difficult to differentiate which independent variable (isolation or group housing) is resulting in the greater impact on cocaine self-administration.

For most of the studies described in this commentary, the inclusion of a social animal was used as an independent variable (for example, to study the negative consequences of social aggression or the positive consequences of access to a social reward or modeling another animal during ongoing drug self-administration). Social status can also be used as a dependent variable, especially if there are known behavioral or neuropharmacological differences associated with social status. In this section, I will briefly explore predispositions to social status (that is, measures taken prior to the formation of social groups) and the consequences of long-term social housing on subsequent cocaine self-administration. In one of the early studies in rodents, Schenk *et al.*^42^ noted that living in social groups decreased acquisition to cocaine self-administration (see 5 for review). Over the last 20 years, the influence of social housing and social status on cocaine self-administration has been extended to nonhuman primate models^22,43–45^. As noted in the other sections, there are excellent reviews of this area (for example, 33,46), so rather than provide an extensive literature on the topic, I will only highlight some important independent and dependent variables for researchers to consider.

These studies use same-sex social groups of female and male cynomolgus monkeys. These monkeys form linear social hierarchies, based initially on the outcome of agonist interactions; the #1-ranked monkey in the pen wins all encounters, the #2-ranked monkey wins fights with all the other monkeys except the #1-ranked monkey, and so on. In social groups of four monkeys per pen, the #4-ranked monkey wins no fights, gets groomed the least, and is a well-characterized model of chronic social stress^47^. For the purposes of this commentary, I’d like to make three observations for future research projects.

When PET imaging was used in drug-naïve and individually housed monkeys, baseline measures of dopamine (DA) D2/D3 receptors were not predictive of future social rank in males^43^ or females^44^. However, for both sexes, becoming dominant resulted in similar increases in D2/D3 receptors in the striatum; but the consequences of these brain changes on vulnerability to cocaine self-administration were opposite^43,44^. After acquisition of cocaine self-administration in dominant and subordinate male monkeys, continued exposure to cocaine eventually resulted in similar rates of cocaine self-administration under an FR schedule of reinforcement and similar measures of D2/D3R availability^48^. Thus, a mechanism that appeared to mediate initial vulnerability in male monkeys (social rank-related differences in D2/D3 receptor availability) was not different during maintenance. However, changing the environmental context in which cocaine was available from a simple FR schedule of reinforcement to a concurrent choice procedure in which monkeys could choose between different doses of cocaine and food reinforcement resulted in subordinate monkeys showing greater sensitivity to cocaine reinforcement^49^. Thus, the contingencies used to study self-administration interact with social status.

Although both dominant male and dominant female monkeys showed similar increases in D2/D3 receptor availability as a result of their social rank, dominant males were protected compared with subordinate males^50^ while dominant females were more vulnerable than subordinate females^44^. Thus, social hierarchy produced sex-dependent differences in vulnerability to cocaine self-administration, despite evidence of similar neurobiological changes associated with DA receptor function. This suggests that there are potential sex differences in the relationship between DA receptors and vulnerability to cocaine abuse and that other mechanisms, beyond DA, should be examined. Some mechanisms currently under investigation include kappa opioid receptors^51,52^. This is another example of the preclinical research findings driving potential clinical research questions.

The final point for this section has to do with the influence of schedule of reinforcement used to study cocaine self-administration and the impact on treatment outcomes. If one is interested in questions related to sensitivity to the reinforcing effects of cocaine (that is, are there sex or social rank differences or both in the lowest dose of cocaine that functions as a reinforcer?), simple schedules of reinforcement, such as FR schedules, are appropriate. However, if one is interested in testing hypotheses related to treatment outcome, more complex schedules, such as concurrent schedules of cocaine vs. a non-drug (typically food, but future studies involve access to a social partner!) alternative, are needed^53^. Of particular relevance to this review is the incorporation of social status in understanding treatment outcomes. One could argue that many of the treatment failures observed in clinical trials are due to the multiplicity of factors that impact potential treatment outcomes, including social factors. Incorporating social variables and identifying potential biobehavioral markers associated with treatment efficacy would represent an animal model using a personalized medicine approach to improve treatment outcomes. Considering published data, we hypothesize that in socially housed males, drugs that increase DA will be more likely to decrease cocaine choice in subordinate monkeys while drugs that decrease DA will be more effective in dominant monkeys. However, in female monkeys, preliminary data suggest that drugs that increase DA neurotransmission appear effective in decreasing cocaine choice in both dominant and subordinate monkeys while decreases in DA tone appear to be more effective in dominant compared with subordinate females (see 45,54,55). Overall, the main points from these studies are the following: (1) in males, drugs that differentially affect DA show rank-related differences; (2) in dominant monkeys, there is clear evidence of sex differences; and (3) no drug uniformly decreases cocaine choice in all four groups. Future research identifying biobehavioral markers associated with efficacy of behavioral and pharmacological interventions is the next step in developing a personalized approach to treatment of cocaine use disorders.

## Conclusions

Two final points are offered to close out this commentary. As noted throughout, there are many ways to incorporate social variables into research and the study design will depend on the questions asked. Four general types of studies incorporating social factors were described: social defeat and neglect, modeling, social reward, and social housing. Ultimately, the ability to do all of these in one setting would yield the most translational findings—this will be the goal of future research. The second point is that although incorporating social variables is clearly the most translational aspect of preclinical research, it will result in greater variability in the data and consequently require more research subjects. This means that the research will be more expensive to conduct and take longer to complete. According to Alvidrez *et al.*^56^, about 90% of R01 grants funded by the NIH and the National Institute on Minority Health and Health Disparities focus on individual-level determinants of health (see also 4). If you believe the inclusion of social variables is necessary for animal research (it is), then funding agencies will have to accept larger budgets and different expectations of productivity. Additionally, all of the studies described in this commentary used same-sex social conditions. This is clearly a limitation, and social housing involving both sexes need to be incorporated into study designs. This too will significantly increase variability.

This commentary began with the observation that animal models have not yet led to effective treatments for cocaine use disorders. The inclusion of social variables into preclinical research will help identify biobehavioral markers that may lead to an individualized treatment approach that more effectively decreases cocaine use. As they relate to non-pharmacological interventions, animal models involving environmental variables—such as punishment (to model incarceration), peers (to model community support), and environmental enrichment (for example, to model the importance of housing, employment, and alternative reinforcers)—will provide valuable data that would be critical for informing policy decisions.
